# 2023 Wildfires in Canada: Living in Wildfire Regions in Alberta and Nova Scotia Doubled the Odds for Residents to Experience Likely Generalized Anxiety Disorder Symptoms

**DOI:** 10.3390/jcm13113234

**Published:** 2024-05-30

**Authors:** Gloria Obuobi-Donkor, Reham Shalaby, Belinda Agyapong, Raquel da Luz Dias, Vincent Israel Opoku Agyapong

**Affiliations:** 1 Department of Psychiatry, Faculty of Medicine, Dalhousie University, Halifax, NS B3H 4R2, Canada; gloria.ob@dal.ca (G.O.-D.); raquell.dias@nshealth.ca (R.d.L.D.); 2 Department of Psychiatry, University of Alberta, Edmonton, AB T6G 2R3, Canada; rshalaby@ualberta.ca (R.S.); bagyapon@ualberta.ca (B.A.)

**Keywords:** anxiety, wildfire, region of residence, Alberta, Nova Scotia

## Abstract

**Background:** Wildfires have become increasingly prevalent in various regions, resulting in substantial environmental and psychological consequences that have garnered increasing attention. **Objective:** This study aims to examine the prevalence of likely Generalized Anxiety Disorder (GAD) and explore the determinants of likely GAD during the wildfires in Alberta and Nova Scotia. **Methods:** Data were collected online through a cross-sectional survey from 14 May–23 June 2023. Alberta and Nova Scotia participants self-subscribed to the program by texting ‘HopeAB’ or ‘HopeNS’ to a short code, respectively. The GAD-7–validated tool was used to collect information on likely GAD. **Results:** This study included 298 respondents while one hundred and twelve respondents lived in a region of Alberta/Nova Scotia affected by the wildfires (37.7%). The prevalence of likely GAD among the respondents was 41.9%. Respondents who lived in a region of Alberta/Nova Scotia recently impacted by the wildfires were twice as likely to experience GAD symptoms (OR = 2.4; 95% C.I. 1.3–4.3). **Conclusions:** The study’s identification of a statistically significant relationship between residing in a wildfire-impacted region and likely GAD shows the association between environmental and psychological well-being. However, the relatively small sample size and self-reported assessment of GAD symptoms may limit the generalizability of the findings. Further research involving a larger sample size delving into potential predictors could facilitate strategies for mitigating the mental health consequences of natural disasters.

## 1. Introduction

Anxiety, a prevalent mental health disorder, has attracted more attention in recent years due to its significant impact on individuals’ overall well-being and daily functioning [1]. Anxiety is characterized by excessive worry, fear, and apprehension, often leading to physical symptoms like restlessness, increased heart rate, and difficulty concentrating [2]. The World Health Organization (WHO) estimates that approximately 264 million individuals globally suffer from anxiety disorders, which makes it one of the most prevalent mental health challenges [3].

The multifaceted nature of anxiety underscores the need for a comprehensive understanding of various factors contributing to its development and exacerbation. One emerging factor contributing to worsening anxiety is wildfires’ escalating frequency and intensity [4,5]. Wildfires, uncontrolled fires that swiftly spread through vegetation and natural landscapes, are now a mounting global concern due to their devastating ecological, economic, and psychological consequences [6]. The environmental impact of wildfires is undeniable, leading to the destruction of natural habitats and altered landscapes [7]. According to systematic reviews, it has been suggested that as many as 40% of individuals who experience a natural disaster like a wildfire may develop stress-related conditions such as anxiety [8]. Moreover, wildfires have profound economic implications, straining firefighting resources and causing property damage worth billions of dollars [9]. Over 5000 fires have been recorded in Canada as of 2 August 2023 [10], with millions spent due to the fire.

As these catastrophic events become more frequent and severe, researchers and mental health professionals are exploring the intricate relationship between anxiety and wildfires [4,11,12]. Additionally, the long-term psychological effects of witnessing the destruction of one’s environment and community can lead to complex mental health conditions like post-traumatic stress disorder (PTSD) [13], depression [14], and anxiety [11]. Moreover, depression and PTSD are the most studied during disasters [15]. A review of quantitative studies including 40 disasters reported various predictors of post-disaster mental health conditions, such as sociodemographic factors and disaster exposure variables. However, the study was limited to PTSD and depression symptoms, while conditions like anxiety need to be examined [16]. In addition, a few literature has shown increased anxiety symptoms following disasters like wildfires and established a relationship between wildfires and the elevated prevalence of anxiety [4,11,12,17]. A study in Korea to assess the impact of 206 survivors’ mental health following the wildfires revealed that 50% expressed anxiety symptoms [4].

The prevalence of anxiety symptoms five years after the 2016 Fort McMurray wildfire was recorded at 42.5% among study participants [5]. A lot of factors may contribute to the mental health effects of individuals who are affected by wildfires. For example, the literature reported that residential location has a remarkable impact on one’s mental health [18]. While wildfires’ physical and economic consequences are well-documented, the psychological toll on individuals, communities, and societies is receiving greater attention. However, studies have focused on PTSD and depression following disasters like wildfire, while the prevalence and risk factors associated with the development of anxiety symptoms are neglected [19]. The experience of living in or near wildfire-prone areas can evoke intense feelings of fear, vulnerability, and uncertainty. The constant threat of evacuation, loss of property, and exposure to smoke and hazardous conditions can trigger or exacerbate Generalized Anxiety Disorder (GAD) symptoms [20].

As researchers delve into the complex interplay between anxiety and the escalating threat of wildfires, a deeper understanding of the other mechanisms involved in increasing the prevalence of psychological effects becomes crucial. This research was conducted against the backdrop of the devastating wildfires that occurred in the provinces of Nova Scotia (NS) and Alberta (AB), Canada, in the year 2023 [21,22]. These wildfires, which gained widespread attention due to their unprecedented scale and impact, provided a unique context for studying such natural disasters’ environmental and psychological implications. The study aimed to comprehensively analyze the wildfire incidents and their aftermath within the two provinces. This study aims to examine the impact of the 2023 wildfires on the prevalence of likely GAD symptoms and potential correlates among residents of Alberta and Nova Scotia, Canada. Consistent with previous studies on mental health conditions post-disaster, we hypothesize that there will be an increased prevalence of GAD symptoms, and geographical location and province will have an impact on GAD symptoms.

## 2. Methodology

### 2.1. Study Setting

The study was conducted in two Canadian provinces: Alberta and Nova Scotia. According to the 2021 census, Nova Scotia province’s population was approximately 969,383 [23]. Alberta is placed fourth as the largest province in Canada and occupies an area of 255,541 square miles [23] and 4,262,635 residents [23]. On 6 May 2023, Alberta declared a provincial state of emergency due to the wildfire, and most residents were also evacuated from their homes [21]. Nova Scotia recorded the largest wildfire in its history, which started on 27 May 2023. The fire destroyed many properties and forced many residents from their homes [22].

### 2.2. Study Design and Institutional Review Board Approval

A data collection approach using a cross-sectional study design was utilized to obtain information from diverse participants within these two provinces. Quantitative data were collected using Research Electronic Data Capture (REDCap 13.7.1) software [24]. Study approval was granted by the University of Alberta Health Research Ethics Committee (Pro00086163) and the Research Ethics Board at Nova Scotia Health (REB file #1028254).

### 2.3. Sample Size Calculation

With a total population of 5,232,018 in both Alberta (~4,262,635 residents) and Nova Scotia (~969,383 residents) according to the 2021 census [23], a 95% confidence interval, and a ±5% margin of error, the sample size required for prevalence estimates for likely GAD will be 385.

### 2.4. Data Collection and Outcome Measures

The study data were collected through an online survey completed by Alberta and Nova Scotia, Canada residents. The study was conducted between 14 May and 23 June 2023. The survey questionnaire included a combination of sociodemographic information, such as gender, age, ethnicity, marital status, employment status, educational status, housing status, mental health status, history of depression or anxiety or a history of receiving psychotropic medications, such as antidepressants and benzodiazepines, and wildfire-related questions like living in a region of Alberta or Nova Scotia that has recently been impacted by the wildfires and the frequency of watching television images about the devastation caused by the wildfires in the two provinces. Subscribers were provided with information about the program and survey, and consent was obtained from those agreeing to participate when they completed and submitted their responses.

Participants self-subscribed to the Text4Hope program and receive daily supportive short message service (SMS) text messages by texting the word “HopeNS” for participants in Nova Scotia and “HopeAB” for participants in Alberta to a short code number (393939). Subscribers could have opted out by texting ‘STOP’ to 393939. The messages adapted cognitive behavioural therapy principles to support individuals during the wildfire. The initial text welcomed subscribers to the program and invited them to complete a voluntary web-based baseline survey to obtain subscribers’ demographic and clinical information. 

An example of a supportive text message is: “There are two days in the week we should not worry about yesterday and tomorrow. That leaves today. Live for today. Thinking of the past or the future can be overwhelming for anyone facing a challenging situation or crisis”.

**Figure 1:**
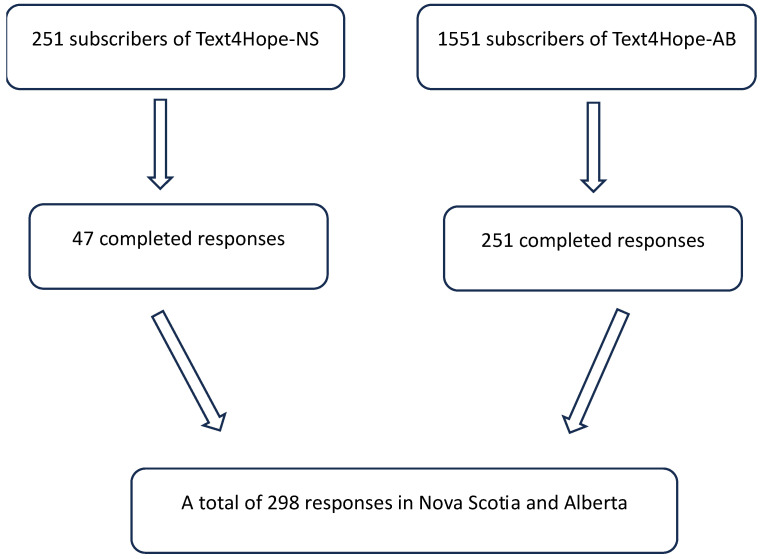
Subscription flowchart.

Figure 1 illustrates the subscription flow chat from 14 May–23 June 2023, in Alberta and Nova Scotia. A total of 251 subscribed to the service from Nova Scotia, with 47 subscribers providing complete responses, yielding a response rate of 18.7%. Similarly, in Alberta, 1551 subscribed to the service, with 251 subscribers providing complete responses, yielding a response rate of 16.2%. Individuals experiencing higher levels of anxiety may have been more likely to drop out of the program, either due to difficulty in completing assessments or a desire to avoid confronting their mental health issues. Conversely, participants with improved mental health over time may have felt less need to continue participating. Technical issues or changes in telephone numbers could also have contributed to attrition. Finally, participants may have faced time constraints or competing priorities that prevented them from completing the assessments. These hypotheses offer possible explanations for the attrition observed in the study.

To assess likely GAD among subscribers, the 7-item brief scale GAD-7 was used [25]. The GAD-7 scale is scored on a 4-point Likert scale, which ranges from “not at all” (0) to “nearly every day” (3) over the last two weeks—the sums of the scores range from 0 to 21 [25]. For analysis purposes, the scores were recategorized into two groups: none to mild anxiety (<10) and moderate to severe anxiety (≥10). The GAD-7 is a valid scale used to screen and assess the severity of anxiety symptoms in both clinical field and research [25,26]. The tool has good test–retest reliability (intraclass correlation = 0.83) with excellent internal consistency (Cronbach α = 0.92) [5,25,27]. The cutoff score was chosen based on established research demonstrating its validity and reliability in clinical and research settings.

### 2.5. Statistical Analysis

The data from the study were analyzed using SPSS for Windows version 28 (IBM Corporation) [28]. Descriptive analysis was showcased in terms of raw numbers and percentages against participants’ gender for all of the demographic, clinical, and fire-related variables. Univariate analysis with chi-squared tests was used to obtain the relationship between likely predictors and subscribers experiencing moderate to severe anxiety symptoms. Statistically significant relationships with variables (*p* ≤ 0.05, two-tailed exact significance) with the likelihood of moderate to severe anxiety on univariate analysis together with predictors which were near significant (0.05 ≤ *p* ≤ 0.1, two-tailed exact significance) were inputted into a logistic regression model. Logistic regression analysis was performed, and strong correlations (Spearman’s correlation coefficient of 0.7 to 1.0 or −0.7 to −1.0 on correlation diagnostics) among predictor variables were excluded. The odds ratios derived from the analysis using binary logistic regression were studied to assess the relationship between each of the variables in the model and the likelihood of respondents experiencing moderate to severe anxiety symptoms, controlling for the other variables in the model. Grossly incomplete surveys were excluded from both data compilation and analysis.

## 3. Results

### 3.1. Descriptive Sample Characteristics

Table 1 illustrates the distribution of sociodemographic and clinical characteristics and wildfire-related variables among the study participants. A total of two hundred ninety-eight (298) respondents completed the baseline survey out of 1802 individuals who subscribed to the program in Alberta and Nova Scotia, giving a response rate of 16.5%.

Table 1 provides information about the distribution of the demographic characteristics by gender of Text4Hope subscribers who completed the baseline survey and summarizes the demographic characteristics of the respondents. As Table 1 displays, most of the respondents were in Alberta (84.2%) aged 59 years and below (225, 75.7%), with the mean age of subscribers being 48.4; most respondents were Caucasian (248, 83.5%), had a postsecondary education (246, 82.8%), were married, cohabiting, or partnered (167, 56.4%), employed (189, 63.6%), and owned homes (200, 67.3%). 

Regarding their clinical history, the majority of participants, 56.6% and 52.9%, indicated that they were diagnosed with depression and anxiety, respectively. In comparison, 63 (21.2%) did not have a mental health condition before the wildfires, with 148 (49.8%) not on any psychotropic medication before the wildfire. On the GAD-7 scale, 41.9% of respondents reported moderate to severe anxiety.

### 3.2. Associations between Sociodemographic, Clinical, and Wildfire Exposure-Related Variables and Moderate to Severe GAD Symptoms

Table 2 illustrates the chi-squared analyses, which show statistically significant (*p* ≤ 0.05) associations between 10 sociodemographic and clinical variables and likely GAD symptoms, including age; employment status; having a history of a depression, anxiety, or a personality disorder, or having no prior mental health diagnosis before the wildfire; being on benzodiazepines or not; no psychotropic medication before the wildfire; living in a region of Alberta or Nova Scotia that the wildfire has impacted; and type of property loss. Respondents’ housing status and a history of alcohol abuse or PTSD/OCD were near significant (0.05 < *p* ≤ 0.1) in the chi-squared analysis.

### 3.3. Predictors of Moderate to Severe GAD Symptoms

The outcomes of the chi-squared analysis were employed to guide the choice of variables for potential inclusion as predictors in a logistic regression analysis. Specifically, twelve of the variables identified via χ^2^ analysis in Table 2 with significant *p*-values (*p* ≤ 0.05) or *p*-values that were approaching significance (0.05 < *p* ≤ 0.1) were entered into the logistic regression model. Table 3 illustrates the results of the Logistic regression model for likely GAD. The comprehensive model, which considered all predictor variables, was statistically significant, χ^2^ (17, N = 248) = 51.35, *p* < 0.01, which suggested that the model could distinguish between respondents with likely GAD symptoms and those who did not report symptoms. Collectively, the model accounted for approximately 18.7% (Cox and Snell R^2^) to 25.2% (Nagelkerke R^2^) of the variance and accurately classified 70.2% of the total cases.

Table 3 shows that only one of the independent variables (living in a region of Alberta/Nova Scotia that the wildfires have recently impacted) made a distinctive statistically significant contribution to the logistic regression model. The odds ratio (OR) for “Live in a region of Alberta/Nova Scotia that the wildfires have recently impacted” was 2.4 (CI of 1.3–4.3), which indicates that respondents who lived in a region of Alberta/Nova Scotia that the wildfires recently impacted were twice as likely to present with likely moderate to severe anxiety symptoms.

## 4. Discussion

The current study’s findings shed light on the profound impact of recent wildfires on the mental well-being of individuals residing in Alberta and Nova Scotia. The research has underscored a significant association between exposure to wildfire-affected areas and heightened anxiety levels among the affected population. Previous research has highlighted the detrimental psychological effects of natural disasters, including wildfires, on individuals’ mental health [5,29,30]. Our study revealed that the prevalence rate of moderate to severe anxiety during the wildfire was 41.9%. This rate is higher than the prevalence of GAD recorded in other studies. For example, the one-month prevalence of GAD after the 2016 Fort McMurray wildfire was 18.0% [31] and 18.7% of college students [32].

Similarly, the Canadian Community Health Survey estimated that approximately 2.5% of Canadians aged 15 years and older reported symptoms compatible with GAD in the previous 12 months and 5% in their lifetime [33]. Another study recorded a slightly higher rate of likely anxiety of 42.5% [5]. Others have recorded varied prevalence in different regions after wildfires that spanned from 27.3% to 46.7% [5,11,34,35]. These findings illustrate significantly elevated rates of various psychiatric disorders in the aftermath of the wildfire compared to the broader Canadian population.

The findings from this study show that most participants reported their gender as female. It is possible that there may have been a higher proportion of female subscribers to the Text4Hope program, leading to a larger pool of potential female participants. Additionally, research has shown that women are more likely than men to seek help for mental health issues and to participate in mental health research studies [36,37].

Our study identified an apparent risk factor for likely moderate to severe anxiety symptoms. Respondents living in a region of Alberta/Nova Scotia recently impacted by the wildfires were twice as likely to express moderate to severe anxiety symptoms. This result accords with other studies highlighting that staying in an area of disaster increases the likelihood of mental distress [30,38]. Various research has shown that Alberta province has been exposed to considerable adversity of natural disasters (wildfires, flooding) [29,30,39], which has exposed residents to mental health consequences. In addition, approximately 40% of individuals living in an area of natural disaster like wildfires are likely to increase their risk of anxiety [8]. This highlights that living in areas of wildfires can involve exposure to adverse events that can precipitate mental health effects. Increases in anxiety symptoms may be explained by the loss of a sense of safety and security due to the recurring threat of wildfires, which may lead to a pervasive state of uncertainty that makes residents constantly worry about their homes, belongings, and loved ones, perpetuating a state of heightened anxiety. Hence, regions affected by wildfires should be provided with accessible and tailored interventions to address anxiety and enhance coping mechanisms.

In our study, most participants were not directly impacted by the wildfires; it is crucial to consider the broader context in which the wildfires occurred. Even individuals living outside the immediate wildfire zone can experience significant anxiety and distress due to various reasons such as media coverage, concern for loved ones, or general feelings of vulnerability to natural disasters [11,32,35,40]. Additionally, the wildfires may have disrupted daily life and routines, causing stress and uncertainty even for those not directly affected. Furthermore, the wildfires may have had a significant psychological impact on individuals living in the broader affected regions, regardless of whether they experienced direct material loss or evacuation [35]. The widespread destruction and disruption during disasters may create a sense of fear and helplessness among affected communities, contributing to increased anxiety levels [30].

Notably, about 62% of our study responders did not live in an area affected by the wildfires in Alberta or Nova Scotia. Hence, moderate to severe anxiety was 42%. A study of the Black Saturday bushfires in Australia reported that most affected communities reported fewer mental health effects among the general population than individuals in highly affected communities impacted by wildfires [38].

The persistent threat of fire-related disasters fosters a chronic state of apprehension [41], especially within impacted areas. Studies of other wildfires have reported that staying in a wildfire region may greatly or mildly affect one’s mental health. For example, the wildfire in California showed that individuals who were directly affected by the fire, including those who suffered property losses or had to evacuate due to flames, and those who merely observed the fires within their community exhibited comparable cognitive impairments [42]. Since our study was conducted during the wildfire era, not much can be deduced from the long-term effect of the wildfire on victims.

The evidence unequivocally indicates that residing in wildfire-prone areas significantly amplifies anxiety levels among affected individuals. The mechanisms underpinning this phenomenon underscore the complexity of the issue and the necessity of addressing mental health alongside physical preparedness and recovery efforts.

## 5. Limitations

While this study sheds light on the link between recent wildfires in impacted regions and anxiety, several limitations warrant consideration. First, the study’s sample might not fully represent the diversity of the population within wildfire-affected communities, considering the approximately 5 million residents in Alberta and Nova Scotia [23]. Insufficient sample size can lead to reduced statistical power, increasing the likelihood of Type II errors and limiting the generalizability of the findings. A larger sample size would allow for more robust statistical analyses. Those mainly affected by anxiety could have been more motivated to participate, potentially skewing the results. Again, moderate to severe GAD symptoms were assessed using a self-reported tool instead of formal diagnostic interviews. The cross-sectional nature of our research precludes causal inferences, and the long-term effect of the wildfire in these regions is unknown. In spite of these limitations, the present results contribute to other natural disaster studies and suggest that living in a region impacted by wildfires affects anxiety levels.

## 6. Conclusions

This study contributes to the growing body of literature highlighting moderate to severe anxiety symptoms of wildfire events in residents of affected regions. The mechanisms underpinning this phenomenon underscore the complexity of the issue and the necessity of addressing mental health alongside physical preparedness and recovery efforts. Swift psychological support initiatives have been shown to mitigate the long-term psychological impacts of disaster-related stressors [43,44]. Collaborative efforts between local authorities, healthcare providers, and mental health organizations are crucial in ensuring the well-being of residents in wildfire-affected areas. By recognizing the emotional toll of living in these areas and implementing targeted interventions, policymakers, mental health professionals, and disaster management officials can effectively support communities, ensuring that the psychological well-being of residents is a central consideration in the face of recurrent wildfires.

## Figures and Tables

**Table 1:** Distribution of demographic and clinical characteristics and wildfire-related items among the study participants.

Variables	Gender			
	Male	Female	Other	Total
	n (%)	n (%)	n (%)	n (%)
**Province**				
Nova Scotia	7 (18.4)	38 (15.0)	2 (33.3)	47 (15.8)
Alberta	31 (81.6)	215 (85.0)	4 (66.7)	250 (84.2)
**Age**				
Median	43.0	51.0	32.0	48.4 (14)
Mean (SD)	44 (14.0)	49.4 (13.7)	34.8 (12.2)	
**Age categories**				
≥60 y	5 (13.2)	67 (26.5)	0 (0.0)	72 (24.2)
50–59	8 (21.1)	71 (28.1)	1 (16.7)	80 (26.9)
40–49	11 (28.9)	50 (19.8)	0 (0.0)	61 (20.5)
≤40 y	14 (36.8)	65 (25.7)	5 (83.3)	84 (28.3)
**Ethnicity**				
Caucasian	27 (71.1)	215 (85.0)	6 (100)	248 (83.5)
Indigenous	1 (2.6)	17 (6.7)	0 (0.0)	18 (6.1)
Asian	4 (10.5)	7 (2.8)	0 (0.0)	11 (3.7)
Black/Hispanic	3 (7.9)	6 (2.4)	0 (0.0)	9 (3.0)
Other	3 (7.9)	8 (3.2)	0 (0.0)	11 (3.7)
**Education level**				
High School or Lower Education	14 (36.8)	37 (14.6)	0 (0.0)	51 (17.2)
Post-secondary Education	24 (63.2)	216 (85.4)	6 (100)	246 (82.8)
**Relationship status**				
Married/Partnered/Common-Law/Cohabiting	18 (48.6)	146 (57.7)	3 (50.0)	167 (56.4)
Single	12 (32.4)	61 (24.1)	2 (33.3)	75 (25.3)
Separated or Divorced	5 (13.5)	33 (13.0)	0 (0.0)	38 (12.8)
Widowed	0 (0.0)	12 (4.7)	0 (0.0)	12 (4.1)
Other	2 (5.4)	1 (0.4)	1 (16.7)	4 (1.4)
**Employment status**				
Employed	28 (73.7)	157 (62.1)	4 (66.7)	189 (63.6)
Unemployed	4 (10.5)	42 (16.6)	2 (33.3)	48 (16.2)
Student	3 (7.9)	9 (3.6)	0 (0.0)	12 (4.0)
Retired	3 (7.9)	45 (17.8)	0 (0.0)	48 (16.2)
**Housing status**				
Own home	22 (57.9)	177 (70.0)	1 (16.7)	200 (67.3)
Renting accommodation	9 (23.7)	52 (20.6)	3 (50.0)	64 (21.5)
Live with family or friend	7 (18.4)	24 (9.5)	2 (33.3)	33 (11.1)
History of mental health diagnosis from a health professional *				
Depression	21 (55.3)	142 (56.1)	5 (83.3)	168 (56.6)
Bipolar Disorder	3 (7.9)	13 (5.1)	0 (0.0)	16 (5.4)
Anxiety	18 (47.4)	134 (53.0)	5 (83.3)	157 (52.9)
Alcohol abuse	2 (5.3)	10 (4.0)	0 (0.0)	12 (4.0)
Drug abuse	4 (10.5)	7 (2.8)	0 (0.0)	11 (3.7)
Schizophrenia	1 (2.6)	2 (0.8)	0 (0.0)	3 (1.0)
Personality Disorder	5 (13.2)	13 (5.1)	2 (33.3)	20 (6.7)
PTSD/OCD	1 (2.6)	15 (5.9)	3 (50.0)	19 (6.4)
ADHD	0 (0.0)	12 (4.7)	2 (33.3)	14 (4.7)
Other	2 (5.3)	2 (0.8)	0 (0.0)	4 (1.3)
No mental health diagnosis	8 (21.1)	54 (21.3)	1 (16.7)	63 (21.2)
History of receiving psychotropic medications *				
Antidepressants	14 (36.8)	97 (38.3)	5 (83.3)	116 (39.1)
Antipsychotics	3 (7.9)	17 (6.7)	1 (16.7)	21 (7.1)
Benzodiazepines	4 (10.5)	11 (4.3)	1 (16.7)	16 (5.4)
Mood stabilizers	7 (18.4)	18 (7.1)	2 (33.3)	27 (9.1)
Sleeping tablets	4 (10.5)	28 (11.1)	1 (16.7)	33 (11.1)
Stimulants for ADHD	0 (0.0)	9 (3.6)	1 (16.7)	10 (3.4)
Other	1 (2.6)	9 (3.6)	0 (0.0)	10 (3.4)
On no psychotropic medication	17 (44.7)	130 (51.4)	1 (16.7)	148 (49.8)
**Received MH counselling in the past year**				
No	19 (50.0)	125 (49.4)	2 (33.3)	146 (49.2)
Yes	19 (50.0)	128 (50.6)	4 (66.7)	151 (50.8)
**Lived in a region of Alberta/Nova Scotia that has recently been impacted by the wildfires**				
No	25 (65.8)	156 (61.7)	4 (66.7)	185 (62.3)
Yes	13 (34.2)	97 (38.3)	2 (33.3)	112 (37.7)
**Evacuation order issued in area of residence**				
Yes	3 (23.1)	24 (24.7)	0 (0.0)	27 (24.1)
No	10 (76.9)	66 (68.0)	2 (100.0)	78 (69.6)
Not applicable	0 (0.0)	7 (7.2)	0 (0.0)	7 (6.3)
**Evacuate from your home due to the recent wildfires in AB/NS**				
No	10 (76.9)	77 (79.4)	1 (50.0)	88 (78.6)
Yes	3 (23.1)	20 (20.6)	1 (50.0)	24 (21.4)
**Loss of property due to the wildfire**				
No	13 (34.2)	94 (37.2)	2 (33.3)	109 (36.7)
Yes	0 (0.0)	3 (1.2)	0 (0.0)	3 (1.0)
Not applicable	25 (65.8)	156 (61.7)	4 (66.7)	185 (62.3)
Kind of property that was lost *				
Home	0 (0.0)	3 (1.2)	0 (0.0)	3 (1.0)
Car	0 (0.0)	1 (0.4)	0 (0.0)	1 (0.3)
No loss	13 (34.2)	93 (36.8)	2 (33.3)	108 (36.4)
**Support from family and friends in relation to the recent wildfire**				
Some-to-absolute support	6 (46.2)	44 (45.4)	1 (50.0)	51 (45.5)
Limited-to-no support	7 (53.8)	53 (54.6)	1 (50.0)	61 (54.5)
**Support from the government of AB/NS in relation to the recent wildfire**				
Some-to-absolute support	3 (23.1)	16 (16.5)	0 (0.0)	19 (17.0)
Limited-to-no support	10 (76.9)	81 (83.5)	2 (100.0)	93 (83.0)
**Support from the Red Cross in relation to the recent wildfire**				
Some-to-absolute support	1 (7.7)	5 (5.2)	0 (0.0)	6 (5.4)
Limited-to-no support	12 (92.3)	91 (94.8)	2 (100.0)	105 (94.6)
**Frequency watching television images about the devastation caused by the recent wildfires in AB/NS**				
Daily	10 (27.0)	93 (36.8)	1 (16.7)	104 (35.1)
About every other day	9 (24.3)	49 (19.4)	1 (16.7)	59 (19.9)
About once a week	5 (13.5)	24 (9.5)	2 (33.3)	31 (10.5)
Less than once a week	6 (16.2)	32 (12.6)	0 (0.0)	38 (12.8)
Not watched TV images of the devastation	7 (18.9)	55 (21.7)	2 (33.3)	64 (21.6)
**Called the Mental Health Crisis line concerning the recent wildfires in AB/NS**				
No	36 (94.7)	250 (98.8)	6 (100)	292 (98.3)
Yes	2 (5.3)	3 (1.2)	0 (0.0)	5 (1.7)
**Likely anxiety**				
Non-to-mild anxiety	17 (60.7)	123 (57.5)	4 (66.7)	144 (58.1)
Moderate-to severe anxiety	11 (39.3)	91 (42.5)	2 (33.3)	104 (41.9)

* Multiple responses provided, MH—Mental Health, ADHD—Attention-deficit/hyperactivity disorder, OCD—Obsessive-Compulsive Disorder.

**Table 2:** Association analysis of demographic, clinical, and wildfire characteristics against likely anxiety.

Variables	None/Mild Anxiety N (%)	Moderate to Severe Anxiety	χ^2^ (df)	*p*-Value
**Province**				
NS	21 (48.8)	22 (51.2)	1.82 (1)	0.18
AB	123 (60.0)	82 (40.0)		
**Age categories**				
≥60 y	46 (75.4)	15 (24.6)	18.4 (3)	<0.001
50–59	33 (53.2)	29 (46.8)		
40–49	36 (66.7)	18 (33.3)		
≤40 y	29 (40.8)	42 (59.2)		
**Gender**				
Male	17 (60)	11 (39.3)	0.29 (2)	0.86
Female	123 (57.5)	91 (42.5)		
Other	4 (66.7)	2 (33.3)		
**Ethnicity**				
Caucasian	128 (61.5)	80 (38.5)	7.24 (4)	0.12
Indigenous	6 (37.5)	10 (62.5)		
Asian	5 (50.0)	5 (50.0)		
Black/Hispanic	3 (42.9)	4 (57.1)		
Other	2 (28.6)	5 (71.4)		
**Education level**				
High School or Lower Education	16 (45.7)	19 (54.3)	2.55 (1)	0.11
Post-secondary Education	128 (60.1)	85 (39.9)		
**Relationship status**				
Married/Partnered/Common-Law/Cohabiting	85 (60.3)	56 (39.7)	3.34 (4)	0.52
Single	31 (51.7)	29 (48.3)		
Separated or Divorced	22 (64.7)	12 (35.3)		
Widowed	4 (40.0)	6 (60.0)		
Other	2 (66.7)	1 (33.3)		
**Employment status**				
Employed	98 (59.8)	66 (40.2)	13.15 (3)	0.004
Unemployed	16 (41.0)	23 (59.0)		
Student	2 (25.0)	6 (75.0)		
Retired	28 (75.7)	9 (24.3)		
**Housing status**				
Own home	103 (62.8)	61 (37.2)	5.60 (2)	0.061
Renting accommodation	31 (52.5)	28 (47.5)		
Live with family or friend	10 (40.0)	15 (60.0)		
History of having mental health diagnosis from a health professional *				
Depression	73 (49.3)	75 (50.7)	11.51 (1)	<0.001
Bipolar Disorder	7 (53.8)	6 (46.2)	0.1 (1)	0.78
Anxiety	68 (48.2)	73 (51.8)	12.99 (1)	<0.001
Alcohol abuse	4 (33.3)	8 (66.7)	3.17 (1)	0.075
Drug abuse	6 (54.5)	5 (45.5)	0.06 (1)	0.81
Schizophrenia	3 (100)	0 (0.0)	2.19 (1)	0.14
Personality Disorder	5 (27.8)	13 (72.2)	7.31 (1)	0.007
PTSD/OCD	7 (38.9)	11 (61.1)	2.93 (1)	0.09
ADHD	6 (50.0)	6 (50.0)	0.34 (1)	0.56
Other	1 (25.0)	3 (75.0)	1.83 (1)	0.18
No mental health diagnosis	47 (79.7)	12 (20.3)	14.83 (1)	<0.001
History of receiving psychotropic medications *				
Antidepressants	55 (53.4)	48 (46.6)	1.58 (1)	0.21
Antipsychotics	8 (42.1)	11 (57.9)	2.15 (1)	0.15
Benzodiazepines	4 (26.7)	11 (73.3)	6.46 (1)	0.01
Mood stabilizers	12 (54.5)	10 (45.5)	0.12 (1)	0.73
Sleeping tablets	16 (50.0)	16 (50.0)	0.98 (1)	0.32
Stimulants for ADHD	4 (66.7)	2 (33.3)	0.19 (1)	0.67
Other	5 (55.6)	4 (44.4)	0.02 (1)	0.88
On no psychotropic medication	78 (64.5)	43 (35.5)	3.97 (1)	0.05
**Received MH counselling in the past year**				
No	73 (62.9)	43 (37.1)	2.12 (1)	0.15
Yes	71 (53.8)	61 (46.2)		
**Live in a region of AB/NS that has recently been impacted by the wildfires**				
No	97 (64.7)	53 (35.3)	6.75 (1)	0.009
Yes	47 (48.0)	51 (52.0)		
**Evacuation order issued for the subscriber area of residence**				
Yes	11 (45.8)	13 (54.7)	1.67 (2)	0.44
No	31 (46.3)	36 (53.7)		
Not applicable	5 (71.4)	2 (28.6)		
**Evacuate from your home due to the recent wildfires in AB/NS**				
No	39 (50.6)	38 (49.4)	1.04 (1)	0.31
Yes	8 (38.1)	13 (61.9)		
**Property lost because of the wildfire**				
No	47 (49.0)	49 (51.0)	1.88 (1)	0.17
Yes	0 (0.0)	2 (100.0)		
**Type of property that was lost**				
Home	0 (0.0)	2 (100)	2.79 (1)	0.95
No loss	47 (49.0)	49 (51.0)	5.33 (1)	0.02
**Support from family and friends in relation to the recent wildfire**				
Some-to-absolute support	22 (54.4)	20 (47.6)	0.58 (1)	0.45
Limited-to-no support	25 (44.6)	31 (55.4)		
**Support from the government of AB/NS in relation to the recent wildfire**				
Some-to-absolute support	8 (53.3)	7 (46.7)	0.2 (1)	0.65
Limited-to-no support	39 (47.0)	44 (53.0)		
**Support from Red Cross in relation to the recent wildfire**				
Some-to-absolute support	1 (20.0)	4 (80.0)		
Limited-to-no support	46 (49.5)	47 (50.5)	1.65 (1)	0.19
**Frequency of watching television images about the devastation caused by the recent wildfires in AB/NS**				
Daily	47 (55.3)	38 (44.7)	0.73 (4)	0.95
About every other day	28 (57.1)	21 (42.9)		
About once a week	16 (64.0)	9 (36.0)		
Less than once a week	19 (59.4)	13 (40.4)		
Have not watched TV images	34 (59.6)	23 (40.0)		
**Called the Mental Health Crisis line in relation to the recent wildfires in AB/NS**				
No	143 (58.4)	102 (41.6)	0.76 (1)	0.38
Yes	1 (33.3)	2 (66.7)		

* Multiple responses provided, df: Degree of Freedom.

**Table 3:** Logistic regression model of study respondents to present with likely anxiety.

Variables	Coefficient	Standard Error	Wald Statistic	df	*p*-Value	Odds Ratio (OR)	95% C.I. for OR	
							Lower	Upper
**Age**								
≥60 years			4.658	3	0.199			
50–59 years	0.588	0.461	1.631	1	0.202	1.800	0.730	4.440
40–49 years	0.028	0.512	0.003	1	0.957	1.028	0.377	2.805
<40 years	0.762	0.492	2.404	1	0.121	2.143	0.818	5.616
**Employment status**								
Employed			4.458	3	0.216			
Unemployed	0.628	0.396	2.521	1	0.112	1.874	0.863	4.070
Student	0.646	0.907	0.508	1	0.476	1.908	0.323	11.287
Retired	−0.532	0.556	0.913	1	0.339	0.588	0.197	1.749
**Housing status**								
Own home			1.379	2	0.502			
Rented	0.050	0.368	0.019	1	0.891	1.052	0.511	2.163
Live with family and friends	0.594	0.509	1.361	1	0.243	1.812	0.668	4.916
**Mental health diagnosis**								
Depression	0.261	0.396	0.434	1	0.510	1.298	0.598	2.818
Anxiety	0.203	0.380	0.287	1	0.592	1.226	0.583	2.579
Alcohol Abuse	1.113	0.689	2.605	1	0.107	3.043	0.788	11.751
Personality Disorder	0.498	0.620	0.646	1	0.422	1.646	0.488	5.546
PTSD or OCD	−0.262	0.607	0.187	1	0.665	0.769	0.234	2.526
No diagnosis	−0.762	0.579	1.733	1	0.188	0.467	0.150	1.451
**Psychotropics Medications**								
Benzodiazepines	1.126	0.671	2.819	1	0.093	3.085	0.828	11.488
No medication for mental health concerns	0.089	0.332	0.072	1	0.788	1.093	0.571	2.095
Live in a region of Alberta/Nova Scotia that has recently been impacted by the wildfires	0.859	0.304	7.985	1	0.005	2.361	1.301	4.284

CI: Confidence Interval.

## Data Availability

The data supporting this study’s findings are available from the corresponding author, Vincent Agyapong, upon reasonable request.
